# Exploring the Role of Caffeine Use in Adult-ADHD Symptom Severity of US Army Soldiers

**DOI:** 10.3390/jcm9113788

**Published:** 2020-11-23

**Authors:** Giada Cipollone, Philip Gehrman, Corrado Manni, Alessandro Pallucchini, Angelo G. I. Maremmani, Laura Palagini, Giulio Perugi, Icro Maremmani

**Affiliations:** 1 PISA-School of Experimental and Clinical Psychiatry, 56100 Pisa, Italy; cipollonegiada@gmail.com (G.C.); corrado3@live.it (C.M.); pallucchini.a@gmail.com (A.P.); angelo.maremmani@uslnordovest.toscana.it (A.G.I.M.); 2 Department of Psychiatry, Perelman School of Medicine at the University of Pennsylvania, Philadelphia, PA 19104, USA; gehrman@upenn.edu; 3 Department of Psychiatry, North-Western Tuscany Local Health Unit, Tuscany NHS, Versilia Zone, 55049 Viareggio, Italy; 4 Association for the Application of Neuroscientific Knowledge to Social Aims (AU-CNS), 55045 Pietrasanta, Lucca, Italy; 5 2nd Psychiatric Unit, Department of Clinical and Experimental Medicine, Santa Chiara University Hospital, University of Pisa, 56100 Pisa, Italy; lpalagini@tiscali.it (L.P.); giulio.perugi@med.unipi.it (G.P.); 6 Vincent P. Dole Dual Disorder Unit, 2nd Psychiatric Unit, Santa Chiara University Hospital, University of Pisa, 56100 Pisa, Italy; 7 G. De Lisio Institute of Behavioral Sciences, 56100 Pisa, Italy

**Keywords:** caffeine, Attention Deficit Hyperactive Disorder (ADHD), Adult-Attention Deficit Hyperactive Disorder (A-ADHD), ADHD symptomatology, United State (US) Army

## Abstract

There is a growing trend of using energy drinks and caffeinated beverages to improve cognitive performance that is widespread and well-studied among children and teenagers with Attention Deficit Hyperactive Disorder (ADHD), but little is known about adult ADHD (A-ADHD). As a consequence, the use of highly caffeinated drinks and their impact on ADHD symptoms are poorly understood. This is especially true in populations where A-ADHD and the use of these beverages are largely represented, such as in military samples. From the All Army Study (AAS) of the Army Study to Assess Risk and Resilience in Service members (STARRS) data, 1,239 A-ADHD soldiers and 17,674 peers without any psychiatric comorbidity were selected. The two groups were compared on: (1) the presence of substance use disorder (SUD) diagnosis both over their lifetime and in the previous 30 days; (2) patterns of alcohol and caffeine use using chi-square analyses. Lastly, the relationship between substance use and severity of A-ADHD symptoms was assessed using Pearson’s correlations. Soldiers with a diagnosis of A-ADHD had a higher prevalence of SUD diagnosis compared to their peers without psychiatric comorbidity. They also tended to use more alcohol, caffeine pills, energy drinks, and other caffeinated drinks. Alcohol use was positively correlated with A-ADHD symptoms; on the contrary, energy drinks, caffeine pills and other caffeinated drinks showed negative correlations with some aspects of A-ADHD symptomatology. The use of caffeinated compounds appears to be increased among military soldiers with ADHD, and they may help reducing A-ADHD symptoms and improve cognitive performance. These results suggest a possible role for caffeine as a potential pharmacological tool in the treatment of adult ADHD.

## 1. Introduction

Attention Deficit/Hyperactivity Disorder (ADHD) is characterized by excessive inattention and/or hyperactivity and impulsivity, as well as executive dysfunction, a broad spectrum of emotional dysregulation with scarce self-control, lack of motivation and functional impairment. In terms of epidemiology, ADHD is estimated to affect 3% to 9% of school-aged children and approximately 4% of adults worldwide [1,2,3,4,5].

Although ADHD is often thought of as a childhood disorder, it often continues beyond adolescence, as shown in long-term controlled follow-up studies. In fact, the disorder seems to persist in a considerable number of adults previously diagnosed with ADHD in childhood [6]. A relevant number of affected children (65%) still manifest symptoms in adulthood [3]. Katzman et al. highlight increasing evidence pointing towards its continuation into adulthood for between 15% and 65% of individuals [7]. Accounting for clinical features, typical motor symptoms of hyperactivity and impulsivity described in the paediatric population may fade later in life, but inattention tends to persist [6]. In fact, in up to 90% of adult ADHD (A-ADHD) individuals, the inattentive symptom pattern is prominently detectable [3]. Like some youth with ADHD, A-ADHD shows correlations with additional cognitive deficits, specifically executive function deficits, including problems encoding and manipulating information and lack of organization and time management skills [8]. A-ADHD prevalence in the US army has been estimated to be around 7.6% and 9.0% depending on the diagnostic method used [9]. Up to date, little has been reported on ADHD in military personnel in the United States of America (USA). However, in the literature some noteworthy data from military contexts of different countries are available. Furthermore, concerns on the possible fitness and eligibility of individuals with A-ADHD have been raised given the demanding and stressful environment of military service, but findings are controversial. Several studies, in fact, have observed negative perceptions of individuals with A-ADHD in the military, lower functional levels, correlations with depression, anxiety, and substance abuse; shorter relationship spans; increased vulnerability to posttraumatic stress disorder; and lower quality of life compared to service members without A-ADHD in a Korean sample [10].

Nevertheless, the discipline of a highly structured training context as in military ones might represent a fertile environment for developing organizational skills for A-ADHD soldiers. Moreover, physical training and exercise might reduce at least some of their symptomatology, as shown in a comparative study of soldiers with and without a history of ADHD in all-volunteer military setting in the USA. Rates of retention, promotion, and mental health-related outcomes during a 5-year period, in fact, showed efficient levels of functioning in those subjects [10]. However, the mentioned study by Noh, Lee and Bahn (2018) was a cross-sectional study with a retrospective chart review, thus not able to determine whether their higher functioning was a direct consequence of participating in duty.

Substance use disorder (SUD) is among the most common comorbid psychiatric conditions in A-ADHD. Substance abuse or dependency is approximately twice as common in individuals with A-ADHD compared to the general population, as a diagnosis of ADHD in childhood leads to a higher vulnerability to substance use and abuse, with an increased risk of developing SUDs among individuals in their 20s and 30s. This reciprocal interaction probably originates from various sources including neurobiological factors and shared genetic risk factors, other comorbid psychiatric disorders, behavioural and temperamental characteristics such as novelty-seeking or impulsivity. In particular, the main drugs of use in the A-ADHD population are alcohol, [11] nicotine, cannabis and stimulant compounds (amphetamines and cocaine) and opiates [12,13] with a duration of illness more than five years for amphetamine (39.5%) and alcohol (56.6%), respectively. As reported by many patients, the use of substances may be an attempt to alleviate or suppress ADHD symptoms, at least temporarily, as postulated in the Self-Medication Hypothesis (SMH) theorized by Khantzian et al. [14,15,16] Khantzian considers the use of addictive drugs as a means of coping with ADHD-related suffering and distress. For example, individuals with ADHD might use cocaine as self-medication to cope with the pattern of emotional distress, inner restlessness and inattention derived from the disease, exploiting the effect of this stimulant on cognition and mood. Thus, the desire for cocaine in A-ADHD with Cocaine Use Disorder (CUD) can be defined as a craving for relief from the cognitive and emotional distress arising from ADHD, negatively affecting the quality of life and social and professional functionality. This type of craving differs from the one usually reported by cocaine users without a diagnosis of ADHD, seeking the recreational and rewarding effects induced by the stimulant. Although controversial, recent findings highlight the efficacy of on-label stimulant treatments in ADHD patients with SUD comorbidity, namely CUD, demonstrating a concomitant improvement in CUD along with ADHD severity, after treatment with Atomoxetine or Methylphenidate. There was a dramatic reduction in CUD symptoms and frequency of use in patients with concomitant ADHD due to a better control of inattentive/emotional symptoms and improved cognitive performance [17].

Little is known about the role of “licit” drugs, such as energy drinks and other caffeinated compounds and their impact on A-ADHD symptomatology and severity. The lack of findings is striking given that worldwide consumption of these products has steadily increased in the past two decades, and they are advertised for their claimed properties of increasing energy and athletic performance and supporting weight loss [18]. These beverages are characterized by high concentrations of caffeine as the main ingredient, as well as vitamins, herbal supplements, sweeteners, and additional stimulants such as taurine, ginseng, and guarana, augment these concentrations [19,20,21,22].

In contrast, the positive influence of caffeine and derivates on working memory, alertness and, in general, cognitive performance is well-established, with some authors recommending low [23] doses of caffeine in children with ADHD, together with prescribed stimulants as a complementary compound. This implementation seems to work as an adjuvant, amplifying therapeutic effects, but the influence of caffeine on single ADHD clinical features and their severity has not been studied yet [24].

The aims of the present study were:

- to explore the use of caffeine, caffeinated drinks and alcohol among soldiers with A-ADHD in order to cope with the cognitive impairment of their condition in the light of the SMH;
- to investigate the impact of these compounds on ADHD clinical features and symptom severity.

## 2. Methods

### 2.1. Design of the Study

The analyses were conducted using data from the All Army Study (AAS) of the Army Study to Assess Risk and Resilience in Service members (STARRS) database, comprising 674,335 United States (US) Army soldiers recruited in quarterly samples from active duty Army personnel.

The AAS study was designed to assess factors related to suicidal risk in military personnel, since suicide rates in the US military has become the second main cause of death behind combat deaths, after the beginning of the Iraq and Afghanistan conflicts, exceeding demographically-matched civilian rates since 2008 [25,26,27].

### 2.2. Sample

From the All Army Study (AAS) of the Army Study to Assess Risk and Resilience in Service members (STARRS) database we focused on the military personnel deployed in Operation Iraqi Freedom/Operation Enduring Freedom (OIF/OEF; N = 21,499) for matching demographics and for the sake of stressor exposure as all models were adjusted for person-year, sex and race-ethnicity. Of these subjects, we selected 1239 soldiers with A-ADHD and 17,674 peers without evidence of psychiatric disorders taken from the original sample, i.e., scoring negatively for all the psychiatric diseases assessed by the questionnaire. Moreover, the population of A-ADHD subjects was diagnosed in childhood, therefore previous to deployment.

### 2.3. Assessment

The AAS is a self-administered questionnaire that was completed between 2011 and 2013, at relatively random times other than at the very beginning or very end of deployment firstly in 2010–2011 in Iraq, and secondly, in 2012–2013, to a supplemental sample unit of soldiers in Afghanistan.

The survey examines current (i.e., past 30 days) and lifetime presence of eight internalizing disorders: major depressive episode (MDE), posttraumatic stress disorder (PTSD), generalized anxiety disorder (GAD), panic disorder (PD), agoraphobia (AGO), specific phobia (SP), social phobia (SO), and obsessive-compulsive disorder (OCD), attention deficit and hyperactivity disorder (ADHD); as well as one externalizing disorder: substance abuse or dependence (SUB/D). Diagnoses were established without DSM-IV diagnostic hierarchy or organic exclusion rules [27].

To assess A-ADHD symptomatology, the survey used the Adult ADHD Self-Report Scale Screener (ASRS-S) [28]. The full ASRS is an 18-item measure developed as part of the World Health Organization’s Composite International Diagnostic Interview (CIDI) with satisfactory internal consistency (Cronbach’s alpha = 0.92: Cronbach’s alpha = 0.87 for inattentive scale, Cronbach’s alpha = 0.84 for hyperactive/impulsive scale). The 14-item screener was developed to optimize clinical utility and has demonstrated psychometric properties equal to or above those of the full ASRS [29]. The ASRS-S assesses ADHD symptom frequency using a 5-point Likert-type scale, with responses ranging from 0 (never) to 4 (very often). Four of the screener items (Items 1 through 4) assess symptoms from the ADHD inattentive symptom cluster, and two of the items (Items 5 and 6) draw from the hyperactive/impulsivity symptom cluster. The ASRS has been used both in military samples and civilian samples, demonstrating good reliability and diagnostic utility [29,30].

The Composite International Diagnostic Interview (CIDI) screening scales for mental health disorders were used to determine the number of current and lifetime mental health disorders. The CIDI Substance Abuse Module, in particular (CIDI-SAM) assesses tobacco, alcohol, drug and caffeine usage in the past 30 days. This measure was developed for clinical and research use, including epidemiological studies; it has demonstrated adequate validity and reliability [31].

The use of over-the-counter stimulant substances was assessed, including use of energy drinks (e.g., Red Bull, Rockstar, Five Hour, Energy, Monster), other caffeinated drink (coffee, tea, Coke or some other soda); caffeinated gum and caffeine or energy pills (NoDoz, Energize or Zoom). Each of the items were investigated in terms of frequency of use on the following scale: every or nearly every day, 3–4 days a week, 1–2 days a week, less than 1 day a week or never, using the past 30 days as the time frame. In addition, the quantity of daily consumption of cigarettes, caffeinated products and energy and alcoholic drinks was assessed. Quantity of daily use was dichotomized as low (less than three per day) or high (3 or more per-day), corresponding to the intake of up to 400 mg/day of caffeine as recommended by the recently released Dietary Guidelines, Health Canada and the European Food Safety Authority.

For alcohol, the Dietary Guidelines for Americans defines moderate drinking as up to 1 drink per day for women and two drinks for men [32].

Since our sample was mainly represented by male individuals, we chose to apply the two-drink a day limit to all our samples [32].

Furthermore, alcohol use was divided into “any drinking” (i.e., use of beer, wine, wine cooler, shot of liquor, mixed drink per day) and “heavy drinking” (i.e., five or more drinks per day) for each week in the past 30 days.

Finally, none of the data in the analyses included caffeinated alcoholic beverages.

### 2.4. Data Analyses

These analyses focused on AAS participants who met criteria for current A-ADHD who were compared to soldiers without evidence of any psychiatric conditions. Chi-square tests were used to compare these groups on: the presence of SUD diagnosis over the lifetime and in the previous 30 days; alcohol and caffeine use. Lastly correlations between use of substances and severity of A-ADHD symptoms were assessed according to the Spearman correlations. SAS system software (version 9.4) was used for our analyses. In consideration of the exploratory nature of the study, we referred to levels of significance of *p* < 0.05, without operating any correction for multiple comparisons.

## 3. Results

Demographic data are reported in Table 1 with comparable results.

Table 2 shows the association between having a lifetime and in the previous 30-day full SUD disorder diagnosis and the presence of A-ADHD. Both lifetime and previous 30-day diagnosis of SUD were significantly more frequent in soldiers with A-ADHD compared to controls.

Table 3 shows the differences in the use of substances, both in terms of a weekly basis and in the past 30 days between soldiers with A-ADHD and without psychiatric comorbidity. Soldiers with A-ADHD reported significantly greater use of prescribed stimulants, Type 1 and Type 2 alcohol, energy drinks, other caffeinated drinks and caffeine pills than their peers without psychiatric diagnosis. All differences were statistically significant.

Table 4 reports the correlations between caffeine use and A-ADHD symptoms comparing ADHD caffeine users and non-users (not using any caffeine compound nor alcohol). Positive correlations were found between alcohol use and “getting things in order”. Negative correlations were found between alcohol use and “past 6 months-drive faster than other”. In other words, more alcohol use, less frequent driving at a faster speed than others and a lower severity of “getting things in order”. Using energy drinks negatively correlated with “feeling overly active and compelled to do thinks”. Using caffeine negativity correlated with the severity of getting things in order, keeping attention on repetitive work, completing tasks in allotted work, stopping from overdoing things and driving faster than others.

## 4. Discussion

In this sample of US Army soldiers, individuals with A-ADHD displayed a higher proneness to suffer from SUD, scoring higher in every domain of the substance use section of the survey, compared to the those without current a psychiatric diagnosis, as a self-relief tool to alleviate ADHD distress, again supporting the SMH.

As previously mentioned, both children and teenagers with ADHD likely develop an increased tendency towards Energy, Caffeine and Caffeinated drink (ECC) consumption and caffeine misuse, with higher rates of intoxication and dependence compared to their peers [33].

In line with these findings, the same trend seems to remain also in adulthood, as our data might confirm, consistently and with the general higher tendency of patients with ADHD to develop SUD, regardless of the drug involved.

Thus, the goal of this study was to evaluate the hypothesis of a higher tendency towards use of stimulant compounds in adults with ADHD. The findings are consistent with this hypothesis, since soldiers with ADHD consumed more caffeinated beverages compared to their non-ADHD colleagues on a weekly basis.

Significantly, in the military population a high rate of energy drink intake is common, and caffeine has been proved to improve reaction time, vigilance and logical reasoning during extended periods with restricted opportunities for sleep, to help sustain workplace safety and productivity [33]. Since military personnel are frequently heavy caffeine drinkers, in part to cope with a demanding lifestyle and a stressful environment, those with ADHD might need even greater doses of caffeine, given the already increased vulnerability to stress and cognitive imbalance connected to their disease. Furthermore, there were interesting correlations between the use of caffeine pills and some ADHD clinical features, mainly affecting cognitive domains, with less problems keeping attention on repetitive work, completing tasks in allotted time and stopping from overdoing things in pill users. They also seemed to have less of a tendency to drive faster than others, compared to ADHD non-users, thus showing less impulsive behaviour. Conversely, only a few significant correlations were found for energy and other caffeine drinks (less problems feeling overly active and keeping things in order, respectively), maybe due to differences in the pharmacodynamic and pharmacokinetic profiles of these given compounds, together with the percentage of caffeine and other ingredients they contained, which likely affects caffeine absorption and metabolism.

However, it is important to note that R squared values were very small, thereby affecting the strength of these correlations, which did not involve most of ADHD clinical features, but only some of them, as reported.

Besides this, the relationship between ADHD symptom severity and use of caffeine seems to point towards a lower impairment of ADHD symptoms in ECC users, defining an inverse correlation among stimulating compounds (caffeine) and ADHD symptom severity which is not replicated when alcohol use is assessed. In fact, alcohol use seems to worsen clinical features except for fast driving where no differences are detected between caffeine use and alcohol users, as both positively affect ADHD driving capacity with a lesser tendency towards dangerous driving. In particular, a possible explanation for the association between A-ADHD and alcohol use that emerges from our analysis could be found in the search for a “relief from relief” effect, where the excessive noradrenergic activation derived from the intake of caffeinated compounds to improve the impaired cognition is compensated with alcohol due to its sedative and hypnotic properties. Therefore, they may try to counterpoise the caffeine-triggered worsening of insomnia and anxiety with alcohol intake, again following the self-medication hypothesis.

For years, the role of caffeine and, more recently, caffeinated drinks has been strongly debated, in consideration of the risk of developing impulsive behaviours up to aggression, irritability and dysphoric mood, increased heart rate, high blood pressure and seizures, together with possible intoxication and withdrawal, especially during the developmental age.

In contrast, the positive influence of caffeine and derivatives on working memory, alertness and, in general, cognitive performance has always been undeniable, with some authors suggesting low doses of caffeine in ADHD children, together with prescribed stimulants as a complementary compound, amplifying their therapeutic effects [24]. Comparable positive influences on A-ADHD cognitive functioning have been reported, together with the reduced, but still considerable side effects aforementioned [24]. Given the fact that high single doses of caffeine are probably less efficient than low to moderate ones, it seems reasonable to guide future research into using low to moderate doses to assess efficacy, with a decreased impact from side effects on health. Another option to contrast and moderate unwanted effects is to work on caffeine pharmacokinetics: caffeine, in fact, has a half-life of 2.5–4.5 h, but the optimal timing and dose interval for caffeine administration is not well understood yet. Exploring the possibility of using modified-release caffeine tablets to facilitate a more consistent plasma level throughout the day and to avoid high peak plasma levels, which can lead to dose-dependent side effects, would be valuable [24,34]. So far, animal models of mice, genetically manipulated to present a hyperactive phenotype, have showed increased performances after receiving caffeine supplements: despite these encouraging results, still greater efforts are needed to test this hypothesis and refine the use of caffeine in everyday clinical practice.

These soldiers with A-ADHD, in fact, although not being completely representative of A-ADHD in the general population, might be considered as high-functioning, with the disease at a low degree in terms of symptom severity and social impairment. In recent work, Nock and colleagues [35] analysed possible risk factors in the Army STARRS soldiers of developing suicidality and self-injuring behaviours. Despite caffeine being considered a trigger for impulsive behaviours in the general population, these data may highlight the importance of caffeine supplementation for dealing with the everyday cognitive impairment of soldiers with ADHD, therefore sensibly changing their life quality and clinical outcomes. Albeit that further research is needed to confirm this hypothesis in the general population, our evidence may highlight the potential benefits of re-opening a dialogue regarding the use of caffeine in ADHD clinical practice, focusing on the identification of possible and safe therapeutic dosages. Moreover, the use of caffeine may draw a new trajectory with potential implications for the use of other adenosine receptor antagonists in ADHD and other disorders characterized by cognitive impairment.

The major limitation of this study is its absence of information regarding possible current specific treatments for A-ADHD. Regarding the presence of psychiatric comorbidity in A-ADHD soldiers, we decided not to adjust for their existence, because the majority of analyses conducted were Chi-square tests, which do not allow the inclusion of covariates for adjustment. Besides, the control sample was without psychiatric comorbidities, and caffeine tends not to have a positive effect on mood and anxiety disorders.

The AAS source data were collected among military personnel and thus, it may not be perfectly representative of the general population. The survey was designed before the release of DSM-5 and, consequently, the diagnostic criteria in it still refers to the previous edition of the manual, DSM-IVR. Furthermore, this is a retrospective analysis; we cannot infer incidence rates of the diseases we focused on. At the time of filling out the questionnaire, some soldiers had already been deployed; others were still waiting for a call to combat. Therefore, some heterogeneity in our sample cannot be excluded.

Finally, the effects on ADHD symptoms are subjective; therefore, neuropsychological studies are recommended to confirm our results.

## 5. Conclusions

Given the lack of data regarding A-ADHD and the use of ECC, we investigated the role of caffeine and caffeinated compounds on cognition and impulsive behaviour, finding a positive influence of caffeine at variable degrees. Therefore, our data may suggest the use of caffeine as an adjuvant and a promising treatment tool to implement the efficacy of currently prescribed stimulants.

However, more efforts have yet to be made in this field of research to identify the correct therapeutic dosage of caffeine and to lessen the impact of side effects, perhaps by discovering new molecules such as adenosine receptor antagonists.

## Figures and Tables

**Table 1:** Demographics.

		Soldiers without Psychiatric Comorbidity N = 17,674	Soldiers with A-ADHD N = 1239
Age	M ± sd	28.72 ± 7.5	28.56 ± 6.9
Male Gender	N (%)	15,606 (88.3)	1090 (88.0)
Race	N (%)		
White		12,460 (70.5)	900 (72.6)
Black or African American		3022 (17.1)	180 (14.5)
American Indian or Alaskan Native		460 (2.6)	53 (4.3)
Asian		742 (4.2)	46 (3.7)
Pacific Islander		247 (1.4)	12 (1.0)
Other		742 (4.2)	48 (3.9)

**Table 2:** Past-30-day and Lifetime Diagnosis of SUD in subjects with A-ADHD and without any psychiatric comorbidity.

	Soldiers without Psychiatric Comorbidity N = 17,674	Soldiers with A-ADHD N = 1239	χ2	*p*
Previous 30-days SUD diagnosis	714 (4.04%)	211 (17.03%)	515.36	<0.0001
Lifetime SUD diagnosis	2639 (14.93%)	503 (40.60%)	780.16	<0.0001

**Table 3:** Per week substance use in A-ADHD soldiers and those without any psychiatric comorbidity (no use: <1 day a week, yes: ≥1 day a week) in the past 30 days.

	Soldiers without Psychiatric Comorbidity N = 17,674	Soldiers with A-ADHD N = 1239		
	N (%)	N (%)	χ2	*p*
Tobacco ^1^	7926 (46.27)	728 (60.41)	90.38	<0.0001
Alcohol use (type 1) ^2^	5160 (29.86)	500 (41.05)	63.86	<0.0001
Alcohol use (type 2) ^3^	2064 (12.04)	305 (25.10)	172.07	<0.0001
Energy drinks ^4^	7386 (42.06)	736 (59.50)	143.27	<0.0001
Other caffeinated drinks ^5^	13,012 (74.17)	1031 (83.55)	53.81	<0.0001
Caffeine pills ^6^	504 (2.87)	1115 (90.36)	164.63	<0.0001

^1^ Cigarettes, cigars, pipes, snuff or smokeless tobacco; ^2^ Beer, wine, wine cooler, shot of liquor, mixed drink; ^3^ Five or more drinks per day; ^4^ Red Bull, Rockstar, Five Hour, Energy, Monster; ^5^ Coffee, tea, Coke, or other soda; ^6^ NoDoz, Energize or Zoom.

**Table 4:** Spearman correlation coefficients between ADHD caffeine-users and ADHD symptoms.

In the Past 6-Month Period:	Alcohol	Energy	Other	Caffeine
		Drinks	Caffeinated Drinks	Pills
Interfered with work/personal life	0.02	0.01	0.01	0.01
	N = 1042	N = 1086	N = 1148	N = 948
Wrapping up final details	0.04	0.02	−0.03	−0.03
	N = 1057	N = 1101	N = 1163	N = 963
Getting things in order	0.07 *	−0.01	−0.06 *	−0.03
	N = 1057	N = 1101	N = 1163	N = 962
Avoid/delay starting a task	0.03	0.01	0	−0.02
	N = 1057	N = 1101	N = 1163	N = 962
Fidget with sitting for a long time	0.01	−0.02	0.02	−0.04
	N = 1057	N = 1101	N = 1163	N = 962
Feel overly active and compelled to do things	0.02	−0.07 *	0	−0.05
	N = 1058	N = 1102	N = 1163	N = 963
Remembering appointments	−0.01	−0.01	−0.05	−0.04
	N = 1058	N = 1101	N = 1163	N = 962
Careless mistakes on boring projects	−0.01	−0.04	−0.02	−0.02
	N = 1057	N = 1100	N = 1163	N = 962
Keeping attention on repetitive work	0	0	0.01	−0.07 *
	N = 1058	N = 1102	N = 1164	N = 963
Completing tasks in allotted time	0.06	−0.04	−0.03	−0.07 *
	N = 913	N = 941	N = 984	N = 891
Prioritizing work	−0.01	−0.01	−0.05	−0.02
	N = 1057	N = 1101	N = 1163	N = 962
Stopping from overdoing things	−0.05	−0.05	−0.03	−0.11 **
	N = 1053	N = 1097	N = 1158	N = 959
Past 6 months driving faster than others	−0.11 **	0.01	0.03	−0.09 **
	N = 1055	N = 1099	N = 1161	N = 960

* *p* < 0.05, ** *p* < 0.001.
